# Supplementary material for: Molecular detection of extended spectrum β-lactamase genes in Escherichia coli clinical isolates from diarrhoeic children in Kano, Nigeria
Source: PLoS One. 2020 Dec 3;15(12):e0243130. doi: 10.1371/journal.pone.0243130 (PMC7714196; doi:10.1371/journal.pone.0243130)
Supplement: Attachment — Submitted filename: Response to reviewers.docx [file pone.0243130.s002.docx]

Dear Editor,

We would like to thank the reviewers for their careful consideration of our manuscript. We have modified the paper according to their suggestions, and detail responses follow:

**Reviewer#1**

*1.- Not all TEM variants are ESBL. Therefore, sequencing the complete orf is essential to identify the variant before relating its presence to the ESBL phenotype, especially in strains where only this gene was identified by PCR.*

We agree with the point made by the reviewer, and in order to follow the suggestion we have now sequenced the complete blaTEM gene in the isolates that were positive in the PCR. This is now indicated in material and methods (lines 119-127) and results (lines 136-139).

*2. From the epidemiological point of view it is important the establishment of the variant of each investigated gene and the ST of at least all ESBL isolates.*

We have identified the subtypes of the blaTEM genes identified in this study, but unfortunately the determination of the sequence type of the studied isolates is beyond the scope of the study and we have not been able to do so due to lack of resources.

*Additionally, I strongly recommend discussing the significant difference between the presence of E. coli ESBL in men and women.*

We thank the reviewer for the comment, we have added a comment on gender differences following the reviewer’s suggestions in lines 169-173

**Reviewer#2**

*Line 38: E. coli, diarrhoeagenic E. coli (DEC) --> could you defined it? As far as I know DEC does not exist, maybe EAEC, EHEC or EPEC.*

We appreciate the reviewers comment on this, DEC was designated as a group by Kaper and Nataro, 1998 (Nataro JP, Kaper JB. Diarrheagenic Escherichia coli. Clin Microbiol Rev. (1998)

11:142–201. doi: 10.1128/CMR.11.1.142) and since their pronouncement of DEC as a group, several articles and published books had recognized DEC as bacterial group.

*Lines 42-45: Newer β-lactamase-producing enterobacteriaceae have been isolated from clinical settings in different parts of the world (Bai et al., 2017) carrying factors such as plasmid-mediated cephamycinases, extended spectrum β-lactamases (ESBLs), and carbapenemases (Jacoby & Munoz-Price, 2005). What do you mean?*

We appreciate the comment of the reviewer. We are reviewing the antibiotic resistance trend in the *β*–lactam agents. We highlight how resistance move from penicillinase to *β*-lactamases before the newer *β-*lactamases. We have completed the sequence to clarify our point in lines 136-139.

*Lines 47-48: They are inhibited by classical β- lactamase inhibitors such as clavulanic acid, sulbactam and tazobactam (Pitout, 2013; Reuland et al., 2013). Please upgrade with new inhibitors and combinations*

We appreciate the comment of the reviewer, newer inhibitors now included along with a more updated reference in Line 48

*Line 99: 20% glycerol were cultured onto EMB agar at 37°C for 18-24 hours. Could you define the medium? ingredients or composition?*

Thank you for the comment. EMB medium is a selective/differential enteric media containing eosin and methylene-blue. The full name has been added to the manuscript line 94-95.

*Table 1: Antibiotic Susceptibility 143 Pattern of ESBLs Producing E. coli. curiosity..... what do you think or consider that can be the cause of this dis-match between phe- and geno-type. did you have some false negative??*

The phenotypic identification of the ESBLs producing isolates was conducted in Nigeria, due to some challenges beyond our control, we could not recover 8 of the phenotypic ESBLs positive when the isolates were due to be transported to Madrid. Therefore, even though there were 38 ESBL-producing isolates (according to their phenotype) only 30 could be subjected to the PCRs for detection of the genes, and all of them were positive for at least one of them.

*Please correct some errors among both tables (1 and 2), CTX, CIP, STX and MDR not match the n (%). f.ex CTX = 22(73.3) and table 2 sum 23*

We appreciate the reviewer for the comment, we have made corrections on the table.
