# Supplementary material for: Molecular detection of extended spectrum β-lactamase genes in Escherichia coli clinical isolates from diarrhoeic children in Kano, Nigeria
Source: PLoS One. 2020 Dec 3;15(12):e0243130. doi: 10.1371/journal.pone.0243130 (PMC7714196; doi:10.1371/journal.pone.0243130)
Supplement: Attachment — Submitted filename: Response to Reviewer.docx [file pone.0243130.s003.docx]

Dear Editor,

We would like to thank the reviewers for their careful consideration of our manuscript. We have modified the paper, though no new suggestions after the last review.
