# Supplementary material for: Molecular detection of extended spectrum β-lactamase genes in Escherichia coli clinical isolates from diarrhoeic children in Kano, Nigeria
Source: PLoS One. 2020 Dec 3;15(12):e0243130. doi: 10.1371/journal.pone.0243130 (PMC7714196; doi:10.1371/journal.pone.0243130)
Supplement: Attachment — Submitted filename: Response to Reviewer.docx [file pone.0243130.s004.docx]

Dear Editor.

We would like to thank the reviewer for the careful consideration of our manuscript. We have modified the manuscript according to the suggestions and response follow:

Reviewer #3

1. *Line- 87-88: The double disc synergy is not recommended by CLSI to confirm the presence o ESBL enzymes in Enterobacterales.*

We appreciate the comment of the reviewer, the information now corrected to reflect our choice of using the double disc synergy test for more specificity in the confirmation of ESBLs phenotype in *E. coli*.

2*. In table number 1. The Augmentin is a commercial name of amoxicillin-clavulanic acid. It*

*should be changed.*

Thank you for the comment, we have made the correction.

3. *The characterization of CTX-M Beta-lactamases in humans samples must be complemented*

*with DNA sequencing.*

We agree with the submission of the reviewer, we could not go beyond typing blaTEM genes due to financial constraints.
